# Electrodialysis Metathesis for the Production of Potassium Phosphate

**DOI:** 10.3390/membranes15050136

**Published:** 2025-05-01

**Authors:** Shichang Xu, Zhen Zhang, Long Zhang, Lixin Xie, Wen Zhang

**Affiliations:** 1 State Key Laboratory of Chemical Engineering, School of Chemical Engineering and Technology, Tianjin University, Tianjin 300350, China; xu_sc1@tju.edu.cn (S.X.); zhangzhen123@tju.edu.cn (Z.Z.); zhanglong123@tju.edu.cn (L.Z.); 2 Tianjin Key Laboratory of Membrane Science and Desalination Technology, School of Chemical Engineering and Technology, Tianjin University, Tianjin 300350, China

**Keywords:** electrodialysis metathesis, potassium phosphate, ion migration

## Abstract

Potassium phosphate (K_3_PO_4_) is a common inorganic compound with broad applications in agriculture and industry. Although the traditional thermal method of preparing K_3_PO_4_ by reacting phosphoric acid with potassium hydroxide can obtain high-quality products, it consumes a lot of energy and has high costs. This study explores the process of preparing K_3_PO_4_ by Electrodialysis metathesis (EDM). This process uses sodium phosphate (Na_3_PO_4_) and potassium chloride (KCl) as raw materials and can prepare K_3_PO_4_ continuously. Under the optimized conditions (operating voltage of 8 V, 0.35 mol/L Na_3_PO_4_ and 1.05 mol/L KCl in raw flow with the rate of 30 mL/min), the product purity of K_3_PO_4_ reaches more than 97%, the energy consumption is 1191 kW·h/t, and the cost is about 8314 CNY/ton. Compared with traditional methods, EDM has the advantages of low cost, simple operation, and high utilization rate. This study shows that EDM technology has significant potential in preparing K_3_PO_4_.

## 1. Introduction

Potassium phosphate (K_3_PO_4_) is a common inorganic compound widely used in various agricultural and industrial fields, such as agricultural fertilizers, food additives, detergents, catalysts, and extractants [1,2]. Currently, the production of K_3_PO_4_ is simple by reacting thermal phosphoric acid with potassium hydroxide. This method can obtain high-quality products, but its energy consumption is high, resulting in high prices for K_3_PO_4_ products. Therefore, with the growing demand for K_3_PO_4_, developing low-cost methods for the K_3_PO_4_ product is significant.

Based on electrodialysis (ED) [3], electrodialysis metathesis (EDM) is a technology that converts one salt into another salt through the reaction between two different salts [4,5]. EDM can efficiently separate multiple ions, with the advantages of being environmentally friendly, having high purity, and low energy consumption [6,7,8,9]. Its unit component usually consists of four ion exchange membranes (alternating cation and anion membranes) [10], including two desalination chambers and two concentration chambers. The ions in the desalination chamber selectively pass through the membrane and generate the desired products, which are difficult to produce under normal conditions [11].

In recent years, EDM has been widely used in the fields of the conversion of high-value salt resources [12,13], treatment of saline wastewater [14,15,16], preparation of ionic liquids [17], and synthesis of organic compounds [18,19]. In the field of salt conversion, EDM can be used to prepare chlorine-free potash fertilizers (such as KNO_3_ [20], K_2_SO_4_ [21,22], etc.), lithium salts (such as LiNO_3_ [23], CH_3_COOLi [24]), etc., with the advantages of low cost and high purity. However, there is no report on the production of phosphate by EDM.

Therefore, we employ EDM to prepare the K_3_PO_4_ product with high purity and concentration in a continuous operation mode here. The Na_3_PO_4_ and KCl were used as the raw materials. We studied the effects of voltage, flow rate, and feed concentration on ion flux, product concentration, purity, current efficiency, and energy consumption, and optimized the process parameters. This paper provides a new strategy for the green synthesis of phosphate products.

## 2. Experimental Section

### 2.1. Experimental Materials

Potassium chloride and sodium phosphate were purchased from Shanghai Dibo Biotechnology Co., Ltd., Shanghai, China, and sodium chloride and potassium phosphate were purchased from Shanghai Bide Pharmaceutical Technology Co., Ltd., Shanghai, China. All reagents were analytically pure. The experimental water was homemade deionized water (conductivity < 10 μS/cm). YRJ cation exchange membrane (CEM) and YRJ anion exchange membrane (AEM) were purchased from Fujian Yanrun Membrane Environmental Protection Technology Co., Ltd., Quanzhou, China. The effective area of each membrane is 0.0442 m^2^. The spacer thickness is 0.1 cm, and the height of the flow channel is 26 cm. Their main properties are listed in Table 1.

### 2.2. EDM Equipment

The laboratory-scale EDM system includes a DC power supply, a membrane stack, pumps, and solution tanks.

**Figure 1:**
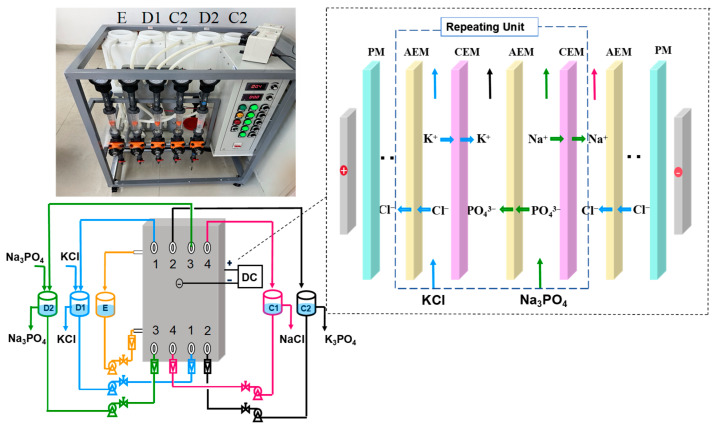
Schematic diagram of the EDM process.

There are 4 repeating units of ion exchange membranes in the whole membrane stack, each consisting of two AEMs and two CEMs. There is a CEM as the electrode membranes (PM) on each side of the electrode plates (Figure 1). The EDM experimental apparatus includes two desalination chambers (Na_3_PO_4_ and KCl), two concentration chambers (K_3_PO_4_ and NaCl), and an electrode chamber. The experiment was operated in a constant voltage operation mode. The KCl water tank is marked D1, the Na_3_PO_4_ water tank is marked D2, the NaCl water tank is marked C1, the K_3_PO_4_ water tank is named C2, and the electrode chamber liquid tank is marked E.

### 2.3. EDM Experimental Procedure

Before the experiment, the prepared KCl and Na_3_PO_4_ raw solutions were added to the desalted water tanks D1 and D2. A certain amount of NaCl solution and K_3_PO_4_ solution was added to the concentration tanks C1 and C2, respectively. A 3% Na_3_PO_4_ solution was added to the electrode tank. Start the power supply, turn on the circulation pump switch, let the solution in the water tank enter each compartment in the membrane stack, and then return to their respective water tanks. The flow rate of the concentration chamber and the desalination chamber is set to 100 L/h. The flow rate of the electrode chamber is also set to 100 L/h. When there are no bubbles in the tube, turn on the DC power supply, adjust the voltage to the set value, and add the KCl and Na_3_PO_4_ raw solutions of equal concentration to the desalted water tanks D1 and D2 at a constant flow rate. The 20 mL samples were taken out every hour. Meanwhile, the pH values of the solutions were measured for the desalination chamber and concentration chamber.

### 2.4. Analysis and Calculation

#### 2.4.1. Concentration Analysis Methods

The concentration of K⁺ ions was determined using the volumetric method with the tetraphenylborate quaternary ammonium salt reagent [25]. The concentration of Cl⁻ ions was measured using the silverometric method [25]. The concentration of PO_4_^3^⁻ ions was determined using the phosphomolybdate quinoline gravimetric method [26]. The concentration of Na⁺ ions was calculated using charge conservation, as shown in Equation (1):

(1) $$C_{{Na}^{+}}=3C_{{{PO}_{4}}^{3-}}+C_{{Cl}^{-}}-C_{K^{+}}$$

#### 2.4.2. Parameter Calculation Methods

1. Ion flux;

Ion flux refers to the number of ions passing through a membrane per unit area per unit time (Equation (2)).

(2) $$J_{ion,Ci}=\frac{\int_{i-1}^{i}cdt\times (V_{i}+V)+(c_{i}-c_{i-1})\times V_{c}}{N\times s\times t}$$

In the equation, *J_ion_*_,*Ci*_ is the ion flux in the concentration chamber, mol/(m^2^ h); *c* is the ion concentration at equilibrium, mol/L; *t* is the time interval between two samplings, 1 h; *i* denotes the *i*-th hour; *V_i_* is the overflow volume of the concentrated product at the *i*-th hour, L; *V* is the sample volume taken each time, 0.02 L; *C_i_* is the ion concentration in the concentration chamber at the *i*-th hour, mol/L; *V_c_* is the volume of the concentration tank, 5.1 L; *N* is the number of the repeating units in membrane stack, 4; *S* is the effective area of each membrane, 0.0442 m^2^.

2. Apparent water flux;

The apparent water flux refers to the increase in the number of water molecules migrating per unit time per unit membrane area in the concentration chamber (Equations (3) and (4)).

(3) $$J_{w,C1}=\frac{\rho_{i,c1}\times V_{i,c1}-V_{i,c1}\times \int_{i-1}^{i}cdt\times M_{NaCl}}{M_{H_{2}O}\times N\times s\times t}$$

(4) $$J_{w,C2}=\frac{\rho_{i,c2}\times V_{i,c2}-V_{i,c2}\times \int_{i-1}^{i}cdt\times M_{K_{3}{PO}_{4}}}{M_{H_{2}O}\times N\times s\times t}$$

In the equation, *J_W_*_,*C*1_ and *J_W_*_,*C*2_ are the apparent water fluxes to C1 and C2 from both membranes forming the compartment, respectively; *ρ_i_*_,*c*1_ and *ρ_i_*_,*c*2_ are the densities of the overflow solution at the i-th hour, g/L; *V_i_*_,*c*1_ and *V_i_*_,*c*2_ are the volumes of overflow solutions, L; *M_NaCl_* is the molar mass of NaCl, 58.5 g/mol; *M_H_*_2*O*_ is the molar mass of water, 18 g/mol; *M_K3PO4_* is the molar mass of K_3_PO_4_, 212.6 g/mol.

3. Energy Consumption;

Energy consumption refers to the amount of electrical energy required to produce 1 ton of product (Equations (5) and (6)).

(5) $$W_{K_{3}PO_{4}}=\frac{U\times I\times t\times 1000}{c\times V\times M_{K_{3}PO_{4}}}$$

(6) $$W_{NaCl}=\frac{U\times I\times t\times 1000}{c\times V\times M_{NaCl}}$$

In the equation, *U* is the stack voltage, V; *I* is the equilibrium current, A; *t* is the equilibrium time, 1 h; *c* is the equilibrium concentration of K_3_PO_4_ or NaCl, mol/L; *V* is the overflow volume of K_3_PO_4_ or NaCl within 1 h, L.

4. Current efficiency;

The current efficiency refers to the percentage of the migrated ions in relation to the total electrical quantity between membranes (Equations (7) and (8)).

(7) $$\eta_{K_{3}PO_{4}}(\%)=\frac{c\times V\times F\times z}{N\times I\times t}\times 100$$

(8) $$\eta_{NaCl}(\%)=\frac{c\times V\times F\times z}{N\times I\times t}\times 100$$

In the equation, *c* is the equilibrium concentration of K_3_PO_4_ or NaCl, mol/L; *V* is the overflow volume of K_3_PO_4_ or NaCl within 1 h, *F* is the Faraday constant, 96,485 C/mol; *z* is ionic chemical valence.

5. Purity.

Purity refers to the proportion of K_3_PO_4_ or NaCl in the product (Equations (9) and (10)).

(9) $$P_{K_{3}PO_{4}}\left(\%\right)=\frac{c\times M_{K_{3}PO_{4}}}{\sum c_{j}\times M_{j}}\times 100\%$$

(10) $$P_{NaCl}\left(\%\right)=\frac{c\times M_{NaCl}}{\sum c_{j}\times M_{j}}\times 100$$

In the equation, *c* is the equilibrium concentration of K_3_PO_4_ or NaCl, mol/L; *c_j_* is the concentration of each component in the mixture (where j refers to K^+^, Na^+^, Cl^−^, PO_4_^3−^), mol/L; *M*_j_ is the molar mass of each component in the mixture (where j refers to K^+^, Na^+^, Cl^−^, PO_4_^3−^), g/mol.

## 3. Results and Discussion

### 3.1. Effect of Voltage

Voltage is a critical parameter in the EDM process and can provide the primary driving force for the mass transfer of ions. Here, we conducted the EDM in constant voltage mode, with the voltages set at 6 V, 7 V, 8 V, and 9 V. The feed flow rate of both Na_3_PO_4_ and KCl solutions was maintained at 30 mL/min, with the feed concentration of Na_3_PO_4_ set at 0.3 mol/L and that of KCl at 0.9 mol/L. The experimental temperature was maintained at 25 °C. The linear flow velocity is 2.67 cm/s.

#### 3.1.1. Ion Flux and Apparent Water Flux

**Figure 2:**
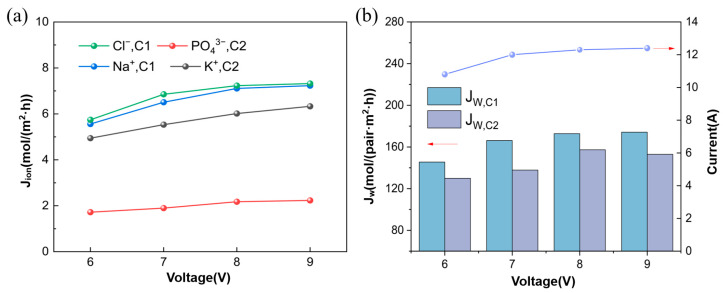
(**a**) Ion flux at different voltages; (**b**) Apparent water flux and current at different voltages (Experimental conditions: feed flow rate of Na_3_PO_4_ and KCl solutions at 30 mL/min, feed concentration of Na_3_PO_4_ at 0.3 mol/L, feed concentration of KCl at 0.9 mol/L, experimental temperature at 25 °C).

As the voltage increases, the ion flux significantly enhances and eventually reaches a plateau, as shown in Figure 2a. The current trend is also shown in Figure 2b. Because the increase in voltage accelerates the ion migration rate, the ion concentration in C1 increases while the ion concentration in the desalination chambers rapidly decreases. In Figure 2a, it can also be observed that J_Na_^+^_, C1_ is approximately equal to J_Cl_^−^_,C1_, while J_K_^+^_,C2_ is about three times that of J_PO4_^3−^_,C2_. J_K_^+^_,C2_ is lower than both J_Na_^+^_,C1_ and J_Cl_^−^_,C1_. Due to the high charge of the phosphate ion, the electrostatic interaction with K⁺ is strong, resulting in a lower flux of K⁺ compared to J_Na_^+^_,C1,_ and J_Cl_^−^_,C1_. The transport mechanism of water through ion exchange membranes involves two key effects. One is driven by concentration gradients, and the other is by the electromigration of hydrated ions under the influence of an electric field [20]. As the voltage increases, both the ion flux and concentration in the concentration chambers significantly increase, leading to a gradual rise in the apparent water flux J_W_, as shown in Figure 2b. The steady-state current under different voltages is shown in Figure 2b. Under the conditions of 6 V, 7 V, 8 V, and 9 V, the current densities are 24.4 mA/cm^2^, 27.1 mA/cm^2^, 27.8 mA/cm^2^, and 28.1 mA/cm^2^, respectively.

#### 3.1.2. Ion Equilibrium Concentration and Energy Consumption

**Figure 3:**
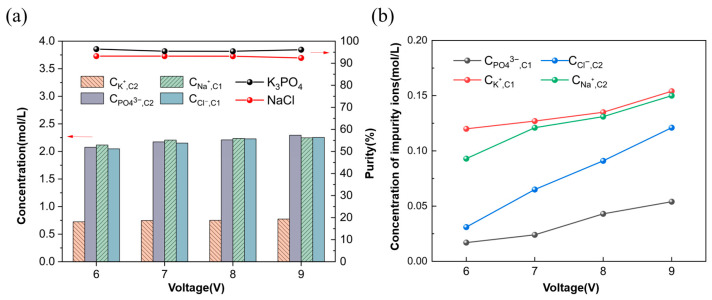
(**a**) Ion concentration and purity under different voltages, (**b**) Impurity ion concentration under different voltages (Experimental conditions: feed flow rates of Na_3_PO_4_ and KCl solutions at 30 mL/min, feed concentration of Na_3_PO_4_ at 0.3 mol/L, feed concentration of KCl at 0.9 mol/L, temperature at 25 °C).

In Figure 3a, as the voltage increases, the ion concentration in the concentration chambers C1 and C2 increases significantly. That is because as the ion flux increases, more ions migrate to the concentration chamber, increasing ion concentration. In the same compartment (C1), C_Na_^+^ and C_Cl_^−^ are slightly different. The selectivity of the ion exchange membrane is not 100%, resulting in phosphate ions and potassium ions mixing into the concentration chamber C1, thus affecting the purity of the product. C_K_^+^ and C_PO4_^3−^ in C2 are not completely consistent with 3:1 due to the mixing of C_Na_^+^ and C_Cl_^−^. Figure 3b shows the concentration of impurity ions in each chamber. It can be found that the concentration of K^+^ and Na^+^ cations is significantly greater than that of anions. This is because their ion radius is small, and crossing the ion exchange membrane is easier. Despite the presence of impurity ions, both NaCl and K_3_PO_4_ products maintained a high purity, 93% and 96%, respectively.

**Figure 4:**
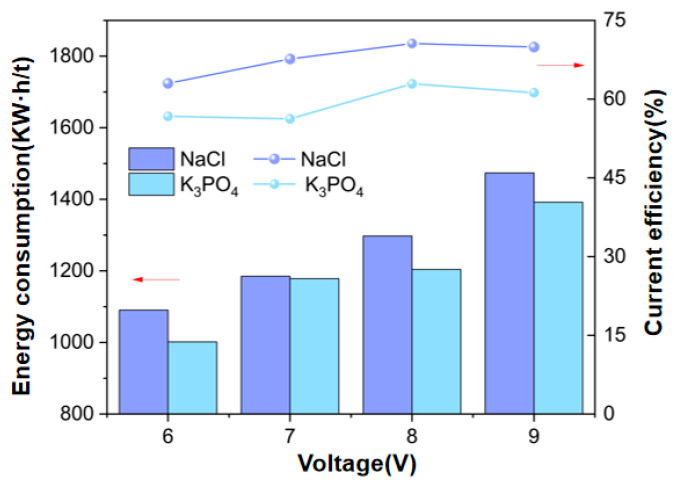
Energy consumption and current efficiency at different voltages (Experimental conditions: feed flow rate of Na_3_PO_4_ and KCl solutions at 30 mL/min, feed concentration of Na_3_PO_4_ at 0.3 mol/L, feed concentration of KCl at 0.9 mol/L, experimental temperature at 25 °C).

In Figure 4, the current efficiency of K_3_PO_4_ and NaCl increases slightly with the voltage increase. This is because when the voltage increases, the driving force for ion migration through the membrane increases, which increases the product concentration in the concentration chamber accordingly. Under 8 V conditions, the current efficiency of K_3_PO_4_ and NaCl solutions reached approximately 63% and 71%, respectively. Voltage is the main factor affecting energy consumption. As the applied voltage increases, energy consumption will also increase. The energy consumption of K_3_PO_4_ increases from 1001 kW·h/t to 1391 kW·h/t with the voltage increase. The energy consumption of NaCl increases from 1090 kW·h/t to 1473 kW·h/t with the increase in voltage, which is quite significant. In the study on the continuous operation of EDM to prepare chlorine-free potassium fertilizer [22], the energy consumption of K_2_SO_4_ and KH_2_PO_4_ was 890 kW·h/t and 1040 kW·h/t, respectively. In this study, the energy consumption of K_3_PO_4_ was above 1100 kW·h/t. This was due to the high resistance of the larger hydration radius ions (e.g., PO_4_^3−^), which resulted in slow ion migration. This requires a higher voltage to drive ion migration, greatly increasing energy consumption.

By analyzing the changes in energy consumption under different voltage conditions and their influence on current efficiency, 8 V is determined to be the optimal operating voltage.

### 3.2. Effect of Feed Flow Rate

This section studied the effect of feed flow rates on the production of potassium phosphate. The feed flow rate ratio of D1 and D2 was fixed at 1:1, and the feed flow rates were set to 27.5 mL/min, 30 mL/min, 32.5 mL/min, and 35 mL/min. The voltage was 8 V, the Na_3_PO_4_ feed concentration was 0.3 mol/L, the KCl feed concentration was 0.9 mol/L, and the experimental temperature was 25 °C. The linear flow velocity is 2.67 cm/s.

#### 3.2.1. Ion Flux and Apparent Water Flux

**Figure 5:**
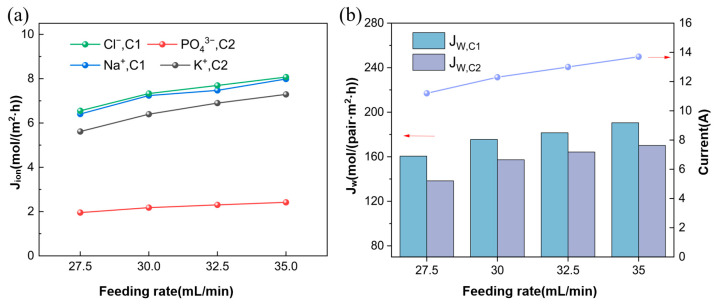
(**a**) Ion flux at different feed flow rates; (**b**) Apparent water flux and current at different feed flow rates (Experimental conditions: voltage of 8 V, feed concentration of Na_3_PO_4_ at 0.3 mol/L, feed concentration of KCl at 0.9 mol/L, experimental temperature at 25 °C).

Figure 5a shows that as the feed flow rate increases, the ion flux in the concentration chamber also increases accordingly. In the continuous operation, the increase in feed flow rate leads to an increase in the number of ions entering the desalination tank (D1, D2) simultaneously, thereby increasing the concentration of the solution. The increase in solution concentration further increases the amount of ions available for migration, ultimately increasing ion flux. The current trend is also shown in Figure 5b. Under the conditions of feed flow rates of 27.5 mL/min, 30 mL/min, 32.5 mL/min, and 35 mL/min, the current densities were 25.3 mA/cm^2^, 27.8 mA/cm^2^, 29.4 mA/cm^2,^ and 31.0 mA/cm^2^, respectively. As the ion flux increases, the water electroosmosis flux driven by ion migration also indicates a significant growth trend, leading to a gradual increase in water flux, as shown in Figure 5b.

#### 3.2.2. Ion Equilibrium Concentration and Energy Consumption

**Figure 6:**
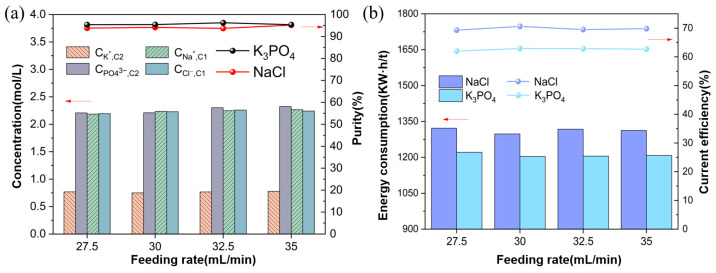
(**a**) Ion concentrations and purities at different feed flow rates; (**b**) Energy consumption and current efficiency at different feed flow rates (Experimental conditions: voltage of 8 V, feed concentration of Na_3_PO_4_ at 0.3 mol/L, feed concentration of KCl at 0.9 mol/L, experimental temperature at 25 °C).

In Figure 6a, as the feed flow rate increases, the ion concentration in the concentration chambers C1 and C2 does not change significantly. This is because the change in ion concentration in the concentration chamber mainly depends on the membrane’s selective permeability and ion migration rate rather than the feed flow rate. Therefore, although the feed flow rate has increased, the ion migration and reaction rate within the system have stabilized, resulting in no significant change in ion concentration. The results show that the purity of K_3_PO_4_ is maintained at about 96%, while the purity of NaCl remains relatively stable at about 94%.

In Figure 6b, as the feed rate increases, the energy consumption and efficiency of K_3_PO_4_ and NaCl remain stable. This shows that changes in feed rate have little effect on process performance. Specifically, the current efficiency of K_3_PO_4_ remains at around 62%, while the current efficiency of NaCl stabilizes at around 70%. At the same time, the energy consumption of K_3_PO_4_ is about 1300 kW·h/t, and the energy consumption of NaCl is about 1200 kW·h/t. These values have almost no significant changes under different feed rate conditions.

### 3.3. Effect of Concentration of the Feed Solution

This section investigates the impact of feed concentration on the production performance of K_3_PO_4_. The experiments were conducted using a constant voltage mode (8 V), maintaining a fixed ratio of 1:3 for the feed concentrations of Na_3_PO_4_ and KCl. The feed concentration of Na_3_PO_4_ were set at 0.25 mol/L, 0.3 mol/L, 0.35 mol/L, and 0.4 mol/L, respectively. Other parameters were kept constant during the experiments: feed flow rate of both Na_3_PO_4_ and KCl solutions at 30 mL/min and experimental temperature at 25 °C. The linear flow velocity is 2.67 cm/s.

#### 3.3.1. Ion Flux and Apparent Water Flux

**Figure 7:**
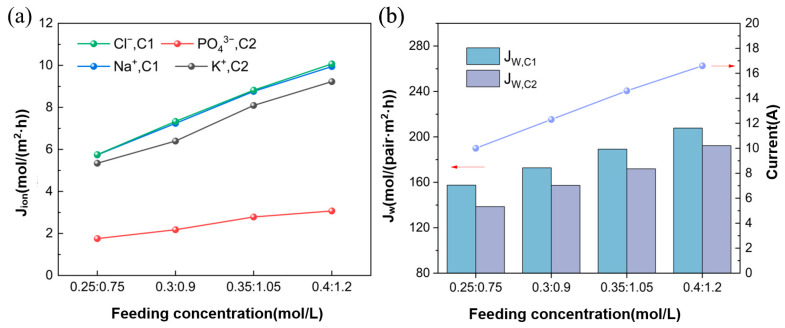
(**a**) Ion flux at different feed concentrations; (**b**) Apparent water flux and current at different feed concentrations (Experimental conditions: voltage of 8 V, feed flow rate of Na_3_PO_4_ and KCl solutions at 30 mL/min, experimental temperature at 25 °C).

Figure 7a,b demonstrates the significant impact of feed concentration on ion flux and apparent water flux. As the feed concentration increases, both ion flux and apparent water flux exhibit a noticeable increase. Compared to the effects of increasing the feed flow rate, the impact of increasing feed concentration is more significant. This result suggests that as the feed concentration increases, the concentration difference between the desalination chamber and the concentration chamber decreases, leading to a reduced ion concentration gradient across the membrane facilitating ion migration. Under the conditions of the raw water concentration of Na_3_PO_4_ being 0.25 mol/L, 0.3 mol/L, 0.35 mol/L, and 0.4 mol/L, the current densities were 22.6 mA/cm^2^, 27.8 mA/cm^2^, 33.0 mA/cm^2^, and 37.6 mA/cm^2^, respectively.

#### 3.3.2. Ion Equilibrium Concentration and Energy Consumption

**Figure 8:**
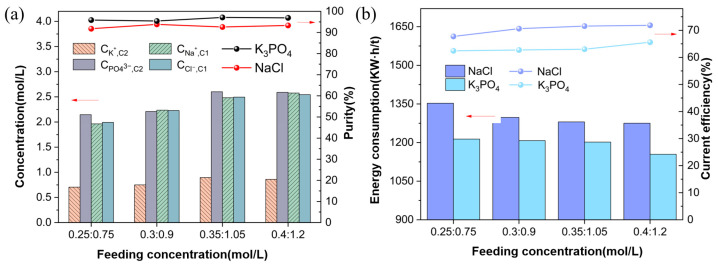
(**a**) Ion concentrations and purities at different feed concentrations; (**b**) Energy consumption and current efficiency at different feed concentrations (Experimental conditions: voltage 8 V, feed flow rate of Na_3_PO_4_ and KCl solutions at 30 mL/min, experimental temperature at 25 °C).

In Figure 8a, as the feed concentration increases, the ion concentrations in the concentration chambers C1 and C2 significantly increase. Increasing the feed concentration enhances the ion flux through the membranes, leading to higher equilibrium concentrations. Additionally, increasing the feed concentration boosts the flux, reducing competition from impurity ions and improving product purity. Under specific feed concentration conditions (Na_3_PO_4_:KCl = 0.35 mol/L:1.05 mol/L), the purity of K_3_PO_4_ reaches its maximum at approximately 97%, while the purity of sodium chloride (NaCl) is about 93%.

In Figure 8b, with the increase in feed concentration, the current efficiency of K_3_PO_4_ and NaCl shows an increase trend. That is because the increase in feed concentration reduces the concentration difference across the membrane, significantly increasing the overflow of C1 and C2 solutions and improving the overall efficiency. Specifically, the efficiency of K_3_PO_4_ increases from 62.4% to 65.6% with feed concentration, and the efficiency of NaCl increases from 67.7% to 71.8% with the increase in feed concentration. With the increase in feed concentration, the energy consumption of K_3_PO_4_ and NaCl does not change much. The energy consumption of K_3_PO_4_ decreases from 1213 kW·h/t to 1154 kW·h/t; the energy consumption of NaCl also decreases from 1352 kW·h/t to 1275 kW·h/t.

Under the condition of Na_3_PO_4_: KCl = 0.35 mol/L:1.05 mol/L in the feed concentration, the product purity is greater than 97%, meeting the national standard GB 25563-2010 [27]. In actual production, using a Mechanical Vapor Recompression (MVR) evaporator to evaporate the solution, the energy consumption for the evaporation of 1-ton water was reported to be about 20–40 kW·h/t [28]. Therefore, for this assessment, we have selected 40 kW·h/t.

We have conducted a comprehensive economic evaluation. Membrane and equipment cost estimates are shown in Table 2, and the total process cost of the process is 619 CNY/t K_3_PO_4_. As shown in Table 3, the production cost of K_3_PO_4_ prepared by EDM is about 8314 CNY/ton. The current market price of K_3_PO_4_ is about 13,500 CNY/ton [29]. That is, the production of K_3_PO_4_ by EDM has a significant economic benefit.

## 4. Conclusions

This study employed the electrodialysis metathesis (EDM) process to produce K_3_PO_4_. The effects of voltage, feed flow rate, and feed concentration on the production performance were investigated systematically.

The voltage is the key factor influencing process performance. Additionally, appropriately increasing the feed concentration can more effectively enhance ion migration and water transport efficiency, improving overall process performance and reducing energy consumption. Under the condition of Na_3_PO_4_: KCl = 0.35 mol/L:1.05 mol/L in the feed concentration, the purity of K_3_PO_4_ reached over 97%, with an energy consumption of 1191 kW·h/t and a cost of approximately 8314 CNY/ton. This study demonstrates the potential of EDM in the production of K_3_PO_4_. Future work could explore the membrane areas and additional repeating units to achieve industrial-scale production. Moreover, EDM shows unique advantages in treating phosphate-containing wastewater by effectively recovering valuable phosphorus resources while reducing environmental pollution, providing an innovative approach for the resource utilization of phosphate-containing wastewater.

## Figures and Tables

**Table 1:** The main parameters used are AEM and CEM.

Membrane Type	Membrane Size/mm^2^	Thickness/mm	Resistance /Ω·cm^2^	Burst Strength /MPa	Recommended Operating Temperature/°C
CEM (YRJCM-I)	400 × 200	0.26	4.0	≥0.25	15–40
AEM (YRJAM-I)	400 × 200	0.17	3.5	≥0.25	15–40

**Table 2:** Estimation of continuous EDM process cost.

Items	K_3_PO_4_	Remarks
Membrane area, m^2^	0.08	
Repeating units	4	
Process capacity, kg K_3_PO_4_/year	737	Annual operation Days = 300
Membrane life and amortization of the peripheral equipment, years	5	
Cation exchange membrane price, CNY/m^2^	500	
Anion exchange membrane price, CNY/m^2^	1000	
Membrane cost, CNY	480	
Stack cost, CNY	720	1.5 times membrane cost
Peripheral equipment cost, CNY	1080	1.5 times stack cost
Total investment cost, CNY	2280	
Amortization, CNY/year	456	
Total process cost, CNY/t K_3_PO_4_	619	

**Table 3:** Costs required for producing 1 ton of K_3_PO_4_.

Type	Name	Consumption	Specific Value (CNY/ton)	Value (CNY)
Material consumption	KCl	1.054 ton	3200	3162
	Na_3_PO_4_	0.773 ton	3200	2474
Electric consumption	EDM(K_3_PO_4_)	1191 kW·h/t	0.7	834
	EDM(NaCl)	1285 kW·h/t	0.7	896
	MVR(K_3_PO_4_)	184 kW·h	0.7	129
	MVR(NaCl)	236 kW·h	0.7	165
Equipment consumption	EDM process cost	1 ton	619	619
Total				8314

## Data Availability

The original contributions presented in the study are included in the article; further inquiries can be directed to the corresponding authors.

## References

[1] Tan D.-Z., Zhang Q.-Y., Chen T.-T., Fan W.-J., Li Y.-N. (2025). Preparing Potassium Dihydrogen Phosphate Using Waste Phosphoric Acid: A Green Chemistry Experiment. J. Chem. Educ..

[2] Kirish K., Vlasov P., Dmitrevskii B. (2020). Production of Potassium Phosphate by a Conversion Method. Fibre Chem..

[3] Xu S., Zhao H., Xie L., Wang K., Zhang W. (2023). Study on the Treatment of Refined Sugar Wastewater by Electrodialysis Coupled with Upflow Anaerobic Sludge Blanket and Membrane Bioreactor. Membranes.

[4] Trivedi J.S., Bhadja V., Makwana B.S., Jewrajka S.K., Chatterjee U. (2016). Sustainable process for the preparation of potassium sulfate by electrodialysis and its concentration and purification by a nanofiltration process. RSC Adv..

[5] Chen T., Bi J., Sun M., Liu J., Yuan J., Zhao Y., Ji Z. (2023). Electrodialysis metathesis for high-value resource conversion and recovery: From sustainable applications to future prospects. Chem. Eng. J..

[6] Meng H., Li H., Li C., Li L. (2008). Synthesis of ionic liquid using a four-compartment configuration electrodialyzer. J. Membr. Sci..

[7] Rottiers T., Bruggen B.V.d., Pinoy L. (2017). Synthesis and transport of impurities in electrodialysis metathesis: Production of choline dihydrogen phosphate. J. Membr. Sci..

[8] Gao W., Zhao H., Wei X., Meng X., Wu K., Liu Y. (2022). A Green and Economical Method for Preparing Potassium Glutamate through Electrodialysis Metathesis. Ind. Eng. Chem. Res..

[9] Chen Q., Zhou Y., Ge S., Liang G., Afsar N.U. (2023). Electrodialysis Metathesis (EDM) Desalination for the Effective Removal of Chloride and Nitrate from Tobacco Extract: The Effect of Membrane Type. Membranes.

[10] Wang X., Du Y., Liu J., Xu F., Ji Z., Guo X., Li F., Yuan J. (2022). Modeling and simulation of continuous electrodialysis metathesis process for conversion of Na_2_SO_4_ to K_2_SO_4_. Desalination.

[11] Wang H., Yan J., Song W., Jiang C., Wang Y., Xu T. (2022). Ion exchange membrane related processes towards carbon capture, utilization and storage: Current trends and perspectives. Sep. Purif. Technol..

[12] Han X., Yan X., Wang X., Ran J., Wu C., Zhang X. (2018). Preparation of chloride-free potash fertilizers by electrodialysis metathesis. Sep. Purif. Technol..

[13] Jaroszek H., Dydo P. (2018). Potassium nitrate synthesis by electrodialysis-metathesis: The effect of membrane type. J. Membr. Sci..

[14] Chen Q.B., Li P.F., Wang J., Xu Y., Zhao J. (2022). Reclamation of sulfate-laden wastewater via a hybrid selective nanofiltration and electrodialysis metathesis process. J. Environ. Chem. Eng..

[15] Zhao Y., Wang X., Yuan J., Ji Z., Liu J., Wang S., Guo X., Li F., Wang J., Bi J. (2022). An efficient electrodialysis metathesis route to recover concentrated NaOH-NH_4_Cl products from simulated ammonia and saline wastewater in coal chemical industry. Sep. Purif. Technol..

[16] Gurreri L., Tamburini A., Cipollina A., Micale G. (2020). Electrodialysis Applications in Wastewater Treatment for Environmental Protection and Resources Recovery: A Systematic Review on Progress and Perspectives. Membranes.

[17] Haerens K., De Vreese P., Matthijs E., Pinoy L., Binnemans K., Van der Bruggen B. (2012). Production of ionic liquids by electrodialysis. Sep. Purif. Technol..

[18] Chai P., Wang J., Lu H. (2015). The cleaner production of monosodium L-glutamate by resin-filled electro-membrane reactor. J. Membr. Sci..

[19] Feng J., Wang Q., Li N., Sun Y., Ma Z., Xu D., Gao J., Wang J., Wang L., Gao X. (2020). Techno-economic evaluation of preparing high-valued TPAOH from its low-cost bromide via electrodialysis metathesis (EDM). Sep. Purif. Technol..

[20] Zhang X., Han X., Yan X., Chen X., Jin Z., Hu X. (2019). Continuous synthesis of high purity KNO_3_ through electrodialysis metathesis. Sep. Purif. Technol..

[21] Liu J., Li D., Xu F., Yuan J., Ji Z., Zhao Y., Li F., Guo X., Wang S. (2021). The efficient conversion of K_2_SO_4_ from Na_2_SO_4_ by continuous electrodialysis metathesis process. Desalination Water Treat..

[22] Sharma P.P., Yadav V., Rajput A., Kulshrestha V. (2018). Synthesis of Chloride-Free Potash Fertilized by Ionic Metathesis Using Four-Compartment Electrodialysis Salt Engineering. ACS Omega.

[23] Wen W.F., Wang J., Zhong C.Y., Chen Q., Zhang W.M. (2022). Direct production of lithium nitrate from the primary lithium salt by electrodialysis metathesis. J. Membr. Sci..

[24] Zhong C.-Y., Lv Y.-P., Wen W.-F., Chen Q., Zhang W.-M. (2022). Sustainable Production of Lithium Acetate by Bipolar Membrane Electrodialysis Metathesis. ACS Sustain. Chem. Eng..

[25] Liu J., Xu F., Yuan J., Ji Z., Zhao Y., Li F., Guo X. (2020). High-value conversion of Na_2_SO_4_ wastewater by a continuous electrodialytic metathesis process: Effects of coexisting ions. J. Membr. Sci..

[26] Jin Y., Li J., Yang B., Zou D., Li W. (2016). Solid-Liquid Equilibria in the Quaternary System K^+^//H_2_PO_4_^2−^, SO_4_^2−^, Cl^−^-H_2_O at 298.2 K and 323.2 K. J. Chem. Eng. Data ACS J. Data.

[27] https://ndls.org.cn/standard/detail/c27e8ed457ab094ec6e941b8f2121a62.

[28] Tiezheng T., Menachem E. (2016). The Global Rise of Zero Liquid Discharge for Wastewater Management: Drivers, Technologies, and Future Directions. Environ. Sci. Technol..

[29] https://www.100ppi.com/mprice/plist-1-4876-1.html.

